# Quality of life in mulptiple sclerosis

**Published:** 2010-11-25

**Authors:** JA Opara, K Jaracz, W Brola

**Affiliations:** * Academy of Physical Education in KatowicePoland; ** Medical University in PoznanPoland; *** District Hospital in KonskiePoland

**Keywords:** amelioration, disability, fatigue, Multiple Sclerosis, Quality of Life, rehabilitation

## Abstract

An overall aim of treatment in  multiple sclerosis is to lower the negative impact of the disease on functioning and quality of life of patients. Therefore, a measurement of functioning and quality of life should be included in  the evaluation of the effectiveness of treatment. In this paper the most commonly used quality of life questionnaires, either generic and specific, were presented. Information about clinical and functional status is useful in the interpretation of the quality of life assessment results. Because of that, instruments for the assessment of  depression, cognitive functions, functional ability and fatigue in multiple sclerosis were also described.

## Quality of Life in MS patients

Quality of Life (QoL) is a multi–dimensional construct which consists of at least three broad domains: physical, mental and social. In the field of medicine researchers and physicians have often used health–related quality of life concept which specifically focuses on the impact of an illness and/or treatment on patients' perception of their status of health and on subjective well–being or satisfaction with life (Jaracz 2003)[1]. We have described the Quality of Life of post–stroke patients and their caregivers in our first report (JMed and Life 2010;3(3):216–220)[2]. The Quality of Life of patients with Multiple Sclerosis is being described in this next review report.

MS can cause a variety of **symptoms**, including changes in **sensation, visual** problems, muscle weakness, **depression**, difficulties with coordination and speech, severe fatigue, cognitive impairment, problems with balance, overheating, and **pain**. MS will cause impaired mobility and **disability** in more severe cases. Multiple sclerosis may take several different forms, with new symptoms occurring either in discrete attacks or slowly accruing over time. Between attacks, symptoms may resolve completely, but permanent neurologic problems often persist, especially as the disease advances. MS currently does not have a cure, though several treatments are available that may slow the appearance of new symptoms.

MS primarily affects adults, with an age of onset typically between 20 and 40 years old, and is more common in women than in men. The course of MS is difficult to predict, and the disease may, at times, either lie dormant or progress steadily. Several subtypes or patterns of progression, have been described. Subtypes use the past course of the disease in an attempt to predict the future course. Subtypes are important not only for prognosis but also for therapeutic decisions. 

Individuals with progressive subtypes of MS, particularly the primary progressive subtype, have a more rapid decline in function. In the primary progressive subtype, supportive equipment (such as a wheelchair or standing frame) is often needed after six to seven years. However, when the initial disease course is the relapsing–remitting subtype, the average time until such equipment is needed is twenty years. This means that many individuals with MS will never need a wheelchair. There is also a more cognitive impairment in the progressive forms than in the relapsing–remitting MS. 

The earlier in life MS occurs, the slower disability progresses. Individuals who are older than fifty when diagnosed are more likely to experience a chronic progressive course, with a more rapid progression of disability. Those diagnosed before the age of 35 years old have the best prognosis. Females generally have a better prognosis than males. However, their Patient–Reported Outcome (shortly PRO) is lower than in men. Women fall in depression and feel fatigue more often; they also suffer from bladder dysfunction more often. A common but often overlooked symptom in MS is sexual dysfunction–in women anorgasmia. Also stressful life events can have strong impact on clinical relapses in women with MS. These are many problems when considering motherhood, referring to pregnancy, child–birth and puerperium (especially breastfeeding) in MS.

QoL measures are suitable as well for outcome measure of new treatment as rehabilitation [3–9]. Subjective factors in QoL in MS patients include perception of symptoms, level of fitness, self–image, satisfaction with family life, work, the economic situation, the interaction with other people, social support and life in general. To the objective factors, we should include the clinical picture of disease, social status, social and living conditions and the number and intensity of social contacts. The scales used to assess the QoL in MS include either subjective or objective indicators, or both[3, 10]. The questionnaire may be completed by the patient in person or by telephone interview, by family members or close persons, by the professional carers and health professionals. The most desirable and reliable is the assessment by the patient himself, especially when the subjects of measurement are subjective aspects of QoL. QoL scales for patients with MS could be divided into universal (general–generic) and specific for the disease (disease–oriented). 

### Generic questionnaires

Among the generic questionnaires used in other disease entities, the assessment of QoL in patients with MS most used are: Medical Outcome Study 36–Item Short Form Health Survey – SF – 36, EuroQol EQ–5D, Sickness Impact Profile (SIP) [9–12], Life Satisfaction Questionaire – LSQ) [13], WHOQOL BREF and Quality of Well–Being Scale – QWBS [11–17].

The above–mentioned questionnaires have been tested in many countries. In the literature there are numerous and detailed data on their validity and reliability –also in relation to MS [18]. The scale of the SF–36 allows the assessment of the eighth areas of QoL during the four weeks preceding the survey, fill it takes about 9 min. It is particularly useful in predicting the course of the disease [19]. The disadvantages include the effect of the lower and upper limit and a relatively low sensitivity to change QoL.

The scale EQ–5D allows the assessment of the fifth areas of health and self–esteem at the time of the study. Filling time is 3 min. Because of the three levels evaluation system EQ–5D is poor sensitive to changes QoL, especially in patients with a score of 5 and over by EDSS. It is intended primarily for managing healthcare – healthcare decision–makers [12].

SIP questionnaire allows the assessment of 12 areas of functioning at the time of study and (in contrast to SF–36 and EQ–5D) is sensitive to the patient's change. The disadvantages of SIP include its length (136 items) which makes the filling time can be up to 30 minutes [13]. LSQ is used to assess the overall satisfaction and satisfaction with the eighth areas of life. The answer to the sixth estimated point scale from very dissatisfied to very satisfied [14]. QWB allows the assessment of mobility, physical activity, social activity and 27 symptoms. The combination of the above categories can identify 43 patient's functional levels. It is recommended that the questionnaire was completed by interview by a person trained for that purpose. Filling time is between 10 – 15 min [17]. 

### Questionnaires specific for MS 

One of the most commonly used scales specific to MS is the Multiple Sclerosis Quality of Life Instrument (MSQoL–54) by Barbara Vickrey and colleagues from the University of California at Los Angeles [20, 21]. This scale is a modification of the SF–36, to which was added 18 questions specific to MS. The tool consists of 52 items grouped in 12 sub–scales and two distinct questions. These are: the impact of the disease, the overall satisfaction with the quality of life, cognitive function, energy, pain, sexual function and social situation. Scale is protected by copyright and its application requires the authors permission each time. Another frequently used tool is the Functional Assessment of Multiple Sclerosis (FAMS), published in 1996 by David Cella and colleagues from Chicago [22]. The original scale consists of 88 questions, was diminutive 44th positional, organized into six subscales. These are: mobility, symptoms and emotional condition, satisfaction, mental activity and fatigue, and family welfare and social–household. In response to each question, the respondent has to choose one of five ability to assess the degree of satisfaction. The Hamburg Quality of Life Questionnaire  in MS – HAQUAMS has been published in 2001 by German authors [23]. It contains 38 questions organized into five domains: mobility of upper limbs, lower limb mobility, social functioning, mood and fatigue / thinking. HAQUAMS largely is based on the SF – 36 and FAMS.

The tool referring only to the subjective indicators of QoL in MS is Quality of Life Index –  QLI, published in 1984 by Carol Ferrans and Marjorie Powers at the University of Illinois at Chicago. There is a generic version of the tool, and six specific varieties, including patients with MS [24]. Questionnaire on QoL in MS patients, like other versions, consists of two parts containing 35 questions each. In the first part of the test answers the questions, how satisfied or dissatisfied with various aspects of their lives. In the second part answers the questions how important it is for him, these spheres of life. The answer to the sixth point scale estimate. The final evaluation for the total scale and four subscales represents the importance of scoring from both parts. Due to the length of and how to record responses, requires very good cooperation from the patient. 

In addition to those mentioned above for the evaluation of QoL in patients with MS also are applied: the Multiple Sclerosis Impact Scale – MSIS–29 [25, 26] and Multiple Sclerosis Quality of Life Inventory – MSQLI. The MSIS–29 is based on self–assessment results of treatment for physical and mental health [27, 28]. MSQLI contains 138 items, organized into 10 generic and specific subscales.[29, 30]. 

### Evaluation of cognitive functions and depression

Scores of QoL are usually supplemented by a study of cognitive function and depression, since these factors significantly affect the sense of quality of life, as well as an important context for the interpretation of test results JZ. Severe cognitive impairment and depressive symptoms may be a contraindication to test PRO. Scoring QoL in patients with MS, especially when it is done for scientific purposes, requires  the measurement of functional status and fatigue, because in addition to depression and cognitive impairment they are the most important determinants of QoL in patients with MS. Following, a review of the most commonly used questionnaires to assess the emotional and cognitive functions is made. 

According to different authors, cognitive dysfunctions are observed in 40 – 65% of patients with MS. In preliminary diagnosis of these disorders the most often used the following tests are: the Benton test, Mini Mental State Examination (MMS), Clock Drawing Test, Rosenbaum vision screening test (Pocket Vision Screener – PVS), Wechsler test [2, 25, 31–37].

In the specific neuropsychological tests commonly used are: Controlled Oral Word Association Test (COWAT), California Verbal Learning Test (CVLT), Digit Symbol Modality Task (SDMT), Delis–Kaplan Executive Function System (D–KEFS), Paced Auditory Serial Addition (PASAT ) [32, 33, 37–39]. The negative impact of cognitive impairment on QoL was demonstrated in a number of studies. Among others in the work of Benito – Leon et al, who used the Mini–Mental scale and the clock drawing test. The results of these tests were negatively correlated with 6 domains of QoL in FAMS [40]. Rivera–Navarro et al evaluated the experiences of caregivers and people with MS. They concluded that social stigma, the lack of work and coming to terms with MS were the greatest issues for the patient, while support from the family network, the relationship that should be established with the patient, the impact of MS on children and the role played by remunerated work were the main dimensions of the disease for the caregiver [41].

Depression in MS is fairly well understood, its prevalence is estimated at 15 to 60% of patients. For the evaluation of depression in MS most frequently applied are: Beck's Inventory, the Hamilton scale, Hospital Anxiety and Depression Scale (Hospital Anxiety and Depression Scale – HADS), Zung scale and the Montgomery–Asberg scale (Montgomery–Asberg Depression Rating Scale – MADRS [31, 42, 43]. Hamilton's scale is more useful for the study of depression in the elderly, while Beck's scale is often used in younger people previously conducted studies clearly indicate that the negative effect of depression on QoL patients. In Benedict et al. studies MS patients reported lower HQOL (p<0.001) and were more likely to be disabled (45% of patients vs. 0 controls). Physical HQOL was predicted by fatigue, depression, and physical disability. Mental HQOL was associated with only depression and fatigue [43]. Lobentanz et al assessed factors influencing QoL in MS. In results most patients were severely disabled; almost half were mildly to severely depressed, suffering from reduced sleep quality and/or fatigue. The multiple sclerosis patients had significantly lower QLI scores than healthy controls. EDSS and SDS (Self–rating Depression Scale) scores were found to be predictors of global QLI score. Regarding the different QLI domains, mean SDS scores remained predictive for all QLI items, while mean EDSS, PSQI (Pittsburgh Sleep Quality Index) and FSS (Fatigue Severity Scale) scores were only predictive for physical domains. In conclusion their study clearly demonstrated that depressive mood is the main factor influencing QOL. The disability status, fatigue and reduced sleep quality have an impact mainly on physical domains of life quality [44].

In Amato et al studies there was a moderate inverse relationship between disability level and the MSQOL–54 physical composite score, and a moderate to strong inverse correlation between depression or fatigue severity and both the physical and mental composite scores. In a stepwise linear regression analysis, depression, fatigue and disability level were confirmed to be significant and independent predictors of quality of life [31].

In the study of quality of life, self–esteem is sometimes extended to pain in many cases. For this purpose the most frequently used are: visual analogue pain scale (Visual Analogue Scale–VAS) and the EQ–5D VAS incorporated into EuroQuol 5D[12, 45, 46]. 

### Global fatigue

Fatigue is one of the most common symptoms of multiple sclerosis and is associated with reduced quality of life. Fatigue in MS has recently been reported in the literature with increasing frequency. This can be defined as uncontrollable apathy, lack of energy or feeling exhausted with no link to depression, or muscle weakness [44, 47–57]. In two thirds of patients with MS it appears as one of the three main symptoms, and patients' opinions of the most troublesome symptoms of the disease. The independence of the degree of fatigue and depression, physical disability, recent studies have confirmed the American and German studies [51, 52].  The fatigue syndrome in patients with MS cannot be evaluated objectively.

That's why for the evaluation of fatigue more than 30 scales has been developed. 

The most frequently used are: the Fatigue Severity Scale – FSS by Krupp et al and the Modified Fatigue Impact Scale (Modified Fatigue Impact Scale – MFIS). FSS is composed of nine items for which the patient responded to 7 point scale estimation. The final result is the arithmetic mean of the scores of all items. The average score of FSS for patients with MS is 6.5 [54]. MFIS is a modification of the scale Fatigue Impact Scale by Fisk et al. It contains 21 items, concerning the impact of fatigue on mental functioning, physical and social. The final result is the sum of points from the scale of individual items [50].

## Evaluation of functional state

Many authors confirmed the importance evaluation of functional status in QoL of MS, although it is smaller than the emotional disturbances. The most common measurement of functional status in patients with MS are the following scales: Expanded or Extended Disability Status Scale – EDSS by Kurtzke, The Scripps Neurologic Rating Scale – SNRS, Barthel Index (BI) and Functional Independence Measure – FIM [22, 58–60]. In EDSS the precise steps are denoted by functional scores which are graded from normal (0) to maximal impairment (5 or 6) for pyramidal, cerebellar, brain stem, sensory, bowel and bladder, visual, cerebral or mental, and other functions. Hobart et al  provided in 2000 a more detailed examination using psychometric methods of Kurtzke's EDSS and FS. Results indicated that the FS measure constructs distinct from each other (intercorrelations = –0.23 to +0. 52) and from the EDSS (correlations = –0.10 to +0.59). Intra–rater, but not inter–rater reproducibility is adequate for group comparison studies. The FS do not satisfy criteria as an eight–, seven– or six–item summed rating scale [61].

In 1999 Cutter et al developed The Multiple Sclerosis Functional Composite – MSFC. The MSFC comprises quantitative functional measures of three key clinical dimensions of MS: leg function/ambulation, arm/hand function, and cognitive function. Scores on component measures are converted to standard scores (z–scores), which are averaged to form a single MSFC score. Time needed for fulfilling all MSFC tests is about 20 min. MSFC components should be administered in the following order: 1. Trial 1, Timed 25–Foot Walk (about 7,62 m), 2. Trial 2, Timed 25–Foot Walk, 3. Trial 1, Dominant Hand, Nine Hole Peg Test (9–HPT), 4. Trial 2, Dominant Hand, 9–HPT, 5. Trial 1, Non–Dominant Hand, 9–HPT, 6. Trial 2, Non–Dominant Hand, 9–HPT, 7. Paced Auditory Serial Addition Test (PASAT) [62].

In 9–HPT the patients is asked to pick up nine pegs into nine holes when time is measured [63, 64]. In 1977, Gronwall introduced the Paced Auditory Serial Addition Test (PASAT) as a measure of the severity of closed head injuries as well as a scale of recovery following a traumatic brain injury [65]. Stimulus presentation rates were adapted for use with MS patients by Rao and colleagues in 1989 [66], and the measure has been widely used in MS studies during the last decade. The PASAT is a measure of cognitive function that specifically assesses auditory information processing speed and flexibility, as well as calculation ability. PASAT is presented on audiocassette tape or compact disk to control the rate of stimulus presentation. Single digits are presented either every 3. (3. PASAT) or every 2. (2. PASAT) and the patient must add each new digit to the one immediately prior to it. The test result is the number of correct sums given (out of 60 possible). 

In Ruddick et al report MSFC scores in patients with relapsing–remitting  MS predicted the level of disability and extended of brain atrophy 6 to 8 years later. The concluded that MSFC scores may prove useful to assign prognosis, monitor patients during early stages of MS, and to assess treatment effects[67]. In Kragt et al study over a period of 2 years in primary progressive multiple sclerosis, the Multiple Sclerosis Functional Composite (MSFC) was less responsive than the Expanded Disability Status Scale (EDSS). The predictive value of neither EDSS nor MSFC was very powerful [68].

## Quality of Life in visual disturbances in Multiple Sclerosis

In Multiple Sclerosis often the visual pathway uses to be impaired. Very early symptom which precede neurological signs is the retrobulbar optic neuritis. In MS patients as well near vision as distance, color and peripheral vision can be disturbed. Many patients are not aware of visual impairment which could cause the delay in diagnose. The assessement of the visual field in patients with MS enables as well early diagnosing as monitoring of the course of disease. 

The most important stage in monitoring the course of disease is the evaluation of quality of life. The assessment of quality of life in MS is well known, even better than in other neurological diseases. Less known is the question of quality of life diminished because of visual field defects in MS. Contemporary possibility of evaluation of quality of life in MS patients include the VFQ–25 questionnaire as a sensitive and useful tool in self-assessing visual function in MS patients. The VFQ–25 consists of 25 questions comprising the 12 sub–scales. They concern the general feeling of health, an overall assessment of vision, ocular pain, near vision, distance vision, social functioning, mental health, difficulty in performing the current role, autonomy, driving, color vision and peripheral vision [69–71].

## Role of comorbidity in MS

In Horton et al study the agreement between self–report and medical records on comorbidity in MS was high (kappa >0.82) for diabetes and hypertension; substantial (kappa = 0.62–0.80) for hyperlipidemia, thyroid disease, glaucoma, and lung disease; moderate (kappa = 0.43–0.56) for osteoporosis, irritable bowel syndrome, migraine, depression, heart disease, and anxiety disorders. Agreement was slight to fair for the remaining comorbidities.[72].
